# Building the capacity of community health workers to support health and social care for dependent older people in Latin America: a pilot study in Fortaleza, Brazil

**DOI:** 10.1186/s12877-021-02477-3

**Published:** 2021-10-02

**Authors:** João Bastos Freire Neto, Gerídice Lorna Andrade de Moraes, Janaína de Souza Aredes, Karla Cristina Giacomin, Luciane Ponte de Melo, Lucas Sempe, Peter Lloyd-Sherlock

**Affiliations:** 1 Fortaleza Municipal Health Secretary, Ceará, Brazil; 2 Universidade Estadual do Ceará. Av. Dr. Silas Munguba, 1700 - Campus do Itaperi Fortaleza, CE (85) 31019800 / 3101-9795. CEP: 60.714.903, Fortaleza, Brazil; 3 Centre for Studies in Public Health and Aging, René Rachou Institute, Fiocruz, Minas Gerais Brazil; 4 Belo Horizonte Municipal Health Secretary, Minas Gerais, Brazil; 5 Brazilian Alzheimer’s Association, Ceará, Brazil; 6 University of East Anglia, School of International Development, Norwich, UK

**Keywords:** Brazil, Community health workers, Training, Older people

## Abstract

**Background:**

Brazil is seeing rapid population ageing, which is leading to new demands on primary health care services. There is a need to develop and assess the effectiveness of new interventions to build the capacity of staff, including community health workers, to meet the needs of groups such as care-dependent older people and their care-givers. This study examines the feasibility of a small training intervention piloted in the Brazilian city of Fortaleza.

**Methods:**

The study evaluated participants’ own assessments of key knowledge and skills related to the needs of care-dependent older people, both before and after the training intervention. It also assessed their capacity to implement a simple screening tool of geriatric risk factors.

**Results:**

The participant self-assessments indicate significant improvements in their perceived knowledge and capacity in responding to the health needs of care-dependent older people. Additionally, participants were able to successfully conduct the home visits and screening for risk factors.

**Conclusions:**

The study demonstrates the feasibility of developing interventions to enhance the capacity of community health workers to meet the needs of dependent older people in countries like Brazil. The evidence of effectiveness, though limited and subjective, provides justification for a larger, formally evaluated intervention. The experience of Fortaleza provides valuable lessons for other cities and countries in the region which are facing similar challenges.

## Background

This paper reviews a pilot training programme for community health workers (CHWs) in the Brazilian city of Fortaleza. The focus of this study was to assess the validity of short training intervention to increase the capacity of CHWs to understand and respond to the health and social care needs of dependent older people living in deprived urban neighbourhoods [1].

Like most LMICs, Brazil is experiencing rapid increases in the number of people in older age groups. Table 1 shows that between 2000 and 2020 the number of people aged 70 or over more than doubled to reach 13 million. Brazil is a large and diverse country, with large socio-economic and demographic disparities between its main regions. The city of Fortaleza is located in the North East of Brazil, a region which, as a whole, has a lower level of economic and human development than the national average. Despite, this it has a similar population age composition to the national average, registering large increases in the number of people aged 70 or over.

**Table 1:** Estimated population aged 70 and over, Brazil and the city of Fortaleza

	2000		2010		2020	
	1000 people	% of total population	1000 people	% of total population	1000 people	% of total population
Brazil	5717	3.3	8597	4.4	12,961	6.1
Fortaleza	70.5	3.5	107.5	4.4	NA	NA

Sources: 29, 31

In Brazil, as in other countries, older adults are more likely to experience chronic health conditions and disabilities, and therefore make more frequent use of health services [2]. These effects are strongly influenced by a range of social determinants, which are especially strong in Brazil’s context of high socio-economic inequality [3]. Brazil has a Unified Health System (SUS) which, in theory, offers universal health care, free at the point of use [4]. SUS has been evaluated as providing good access to health services, with an emphasis on primary health care services and reduced pressure on hospital services [5]. Despite being a national system, SUS is strongly decentralised, with considerable autonomy and scope for policy innovation at the level of local, municipal government [6]. In Fortaleza, as elsewhere in Brazil, this includes a strong focus on family health services, provided by inter-disciplinary teams of health professionals and community health workers operating out of local health centres. There has been no research about the specific roles that these CHWs may be able to play in supporting service provision for older adults.

The World Health Organisation defines CHWs as individuals who perform functions related to healthcare delivery, have less training than professional health workers and are members of the same communities they work in [7]. This is a broad category and it has been observed that the nature and status of this role varies substantially between countries [8].

Historically, in countries like Brazil, they have been mainly trained for specific tasks such as provision of antenatal care or immunisation and rarely hold formal health qualifications. In recent years there has been growing interest in expanding this repertoire of competencies to include emerging health conditions in LMICs, such as non-communicable diseases (NCDs) [9]. Evidence from other countries demonstrates the scope for CHWs to make valuable contributions to programmes to manage chronic conditions, if adequately trained and supported [8].

Since the 1980s CHWs have played had a particularly important role in supporting family-oriented primary health care in Brazil. Fortaleza was at the forefront of these developments, with a dual objective of reducing high rates of infant mortality and generating new opportunities for women whose livelihoods had been hit by a severe drought [10]. CHW responsibilities are wide-ranging and include education, health promotion, maintaining patient records, making regular house-calls, monitoring vaccination uptake and providing referrals. In the tropical context of Fortaleza, a key focus of CHW activity has been to mitigate the effects of infectious diseases, such as seasonal dengue, in poorer neighbourhoods. Though these remain important activities, Fortaleza’s epidemiological profile has followed global trends towards a growing burden of NCDs. For example, in 2017 fewer than 1000 deaths were attributed to infectious disease, compared to over 7000 resulting from either cancer or cardio-vascular disease [11].

CHWs live in the same communities they work in which gives them detailed local knowledge and easy access to households. This, in turn, enables them to perform a bridging role between health care providers and these communities, taking into account local health beliefs and practices [12, 13]. However, it has been observed that CHWs often have a marginal role and limited influence within community health teams [14]. Studies identify a wider set of challenges faced by CHWs in Brazil, including a lack of resources and limited integration with wider health teams [15, 16]. All CHWs receive some general training on recruitment, but does not include specific content related to the health or care needs of older adults. More generally, it is claimed that few resources are invested in training CHWs or providing them with accredited qualifications [13].

The experiences of high-income countries, as well as other parts of Brazil (such as the city of Belo Horizonte) indicate that there may be particular benefits in developing integrated, community-based health and social care interventions for frail older people in deprived neighbourhoods: both to enhance the quality of life of these older people and their families and to encourage more efficient use of available health services [17, 18]. Such interventions are likely to require a significant role for CHWs, and this will require awareness of how health and social care issues affect older people in their communities.

Partly inspired by wider experiences, in 2019 Fortaleza’s Department of Health developed and ran, for the first time, a training intervention to assess CHWs prior knowledge of these issues and to develop their capacity. This paper examines the participants and course leaders’ experiences of the programme, to identify lessons for similar future interventions in Fortaleza and beyond.

The training intervention took place in the city of Fortaleza, the capital of the state of Ceará, located in the Northeast of Brazil. The region is known for its high poverty index and limited health and social services resources infrastructure compared to other parts of the country [19]. Currently there are 126,000 older people registered with by the Family Health Strategy teams (Estratégia Saúde da Família, in Portuguese), representing 48% of the city’s older population. The local health department estimates there is a population of 5600 dependent older people (DOPs), who need basic assistance in daily life activities. In response to this emerging need, the municipal departments of health and social development worked with the Ceará branch of the Brazilian Association for Alzheimer to design and run a pilot training intervention to increase the capacity of CHWs to support home care for DOPs.

The intervention was applied in three community primary care units (PCUs) located in neighbourhoods containing the largest numbers of older people identified as socially and economically vulnerable. Of a total of 76 CHWs were working in these PCUs. All were invited by the PCU manager to take part in the training programme and it was agreed that their line managers would reduce their workloads to compensate for the additional activity. Of these 76 CHW, 72 participated in classroom-based sessions and 58 also participated in the practical home visits element. All participants had completed secondary school and had at least 12 years’ related work experience, 47 were women (with an average age of 47) and 11 were men (average age 44).

Due to the lack of previous training of CHWs on issues relating to dependent older people, the training looked to address a wide set of issues. The content of the training programme also took into account specific contextual issues. For example, the limited development of social services for older people in cities like Fortaleza means that, almost without exception, care-giving is almost performed by family members with little, if any, support from or interaction with outside professionals [20]. The content of the course was geared to enhancing the capacity of CHWs to work with these family care-givers and to be able to perform simple screenings of dependent older people’s status.

The specific course content was adapted from a specialist training programme for primary health care professionals on the health of older people produced in 2018 [21]. It covered five topics: the health of dependent older people; identifying risk situations; essential health and social support for dependent older people; and caregiver support (Table 2). The course content also reflected responses provided by CHWs to a self-evaluation conducted before the training started. This included the CHWs’ assessments of their knowledge about older people’s health and care needs. Only ten CSWs had received any previous training about older people’s health and in no case had this exceed 10 hrs in total. Reflecting CHW responses, the training focussed on helping them to identify potential risk factors for frail older people in home settings, and included preventive strategies involved the use of routine of home visits, as well as basic treatments. Given CSWs’ interest in the wider social context of care-giving, the course sought to enhance their capacity to work with social services, as well as with the family care-givers themselves.

**Table 2:** Content of the classroom-based training sessions

Topic	Content
Health of the Dependent older Person	✓ - Health and functional capacity in later life
	✓ - Activities of Daily Living
	✓ - Who is a dependent older person?
Essential Health Care I	✓ - Hygiene
	✓ - Continence
	✓ - Nutrition
Essential Health Care II	✓ - Transference
	✓ - Mobility
Aggravating Situations I	✓ - Risk Situations
	✓ - Communication with the Family Health Strategy Team
Aggravating Situations II	✓ - Prevention of violence
Social Support	✓ - Basic social support (from Social Work) available to older people
	✓ - Dependent older people and their families
Care-giver Staff Support	✓ - The care-giver and the dependent older person
	✓ - Taking care of the care-giver

The classroom-based component consisted of seven half-day sessions running between March and May 2019, totalling 28 h. These were split into two separate classes to reduce the number of CHWs in each session. The classes were led by doctors and nurses with specialist backgrounds in geriatric and gerontology. A further 12 h of training were dedicated to practical activities, consisting of house visits to DOPs in the locality. This activity was conducted after the classes and was supervised by the nurse in charge of each PCU who already had training relating to dependent older people. The visits were seen as an integral part of the training programme. A total of 216 dependent older people were identified from health centre records, of whom 66.2% were women and with an average age of 81.

To this end, the course applied different methods, including theoretical and practical activities. Following the approach advocated by Ritter et al. (2014), the methodology was participatory and reflective [22]. To link participants’ learning with their work experiences, everyday situations encountered by community health teams were used to develop simulations, role-plays and group discussions. This gave space for participants to express opinions, report their own experiences related to the themes and clarify doubts about how to respond to specific situations. Emphasis was given to fostering dialogue between the trainers and the participants, valuing the knowledge and insights of both [23]. Although the CHWs did not have previous training in this area, many had experiences of dealing with dependent older people and family care-givers in the course of their more general health activities. Following established principles of health professional education, the training aimed to relate technical knowledge, clinical practice, more general experience of participants to a holistic concept of health [23].

## Methods

The small scale of the intervention and limited resources did not permit the application of a formalised evaluation design. Instead, the study draws, to some degree opportunistically, on data that were collected in the course of planning the intervention, applying the practical element of the intervention and assessing participants’ views about the effects the intervention had on their knowledge of specific issues. The participants were informed about the wider application of their contributions to data collection and of their right to withdraw from the study at any moment, as well as about the voluntary aspect of their participation [24]. These were derived from brief structured interviews conducted with staff after the intervention had been completed.^1^ Throughout the training and evaluation, participant anonymity was guaranteed.

One key source of data for this study was a CHW self-evaluation conducted in advance of the training intervention. The aim of this evaluation was to identify existing areas of knowledge and gaps related to the health of dependent older people and how their health and care needs could be best met by in deprived urban settings. This information informed the content of the class-based training sessions and offered a point of comparison for CHW evaluations conducted after the training had been completed. Three months after the completion of the training programme, an additional CHW self-evaluation was conducted. This included the same items as in the first assessment, as well as items about their opinions of the training intervention and their perceptions of how it had affected their related competencies. As part of this second evaluation, CHWs were asked to identify aspects of competencies which they felt remained weak and required further training.

A further source of data was the information CHWs and nurses collected in the course of the home visits which formed part of the formal training programme. They included specific risk factors identified through the screening tool. This was applied by the CHWs and then verified by the trained nurses who accompanied them on the visits. The degree to which these visits were completed and the screening tool implemented can be understood as a useful indication of the validity of the intervention process rather than of outcomes per se*.*

This study design has some limitations in comparison with a more systematic evaluation design developed in advance of the intervention. It had been planned to conduct more detailed interviews with both participants and trainers in the months following the intervention. This was not possible, due to the onset of the COVID-19 pandemic. Fortaleza has been one of the most badly affected cities in Brazil and all potential interviewees have been directly involved in the emergency public health response. It was possible to hold some informal discussions with trainers about the nature of classroom interactions and dynamics. Due to the scant time available to these staff (all of whom are health professionals), this information is limited and their recall may not be entirely reliable. Nevertheless, despite these important limitations, the combined set of available data provides insights about both the intervention process and, to a lesser effect, its outcomes. The value of process evaluation of health interventions has gained recognition in recent years, as a means to assess the fidelity of implementation and the degree to which context-specific interventions may be replicated [25].

## Results

A key fidelity indicator for the implementation of the training programme was the retention of CHWs through the class-based sessions and their effective participation in the practical home visits. Of the 76 CHWs who took part in the classroom-based component, 72 completed the full set of home visits. The four who did not complete this element of the training programme expressed interest in participating, but it was not possible to fit the visits within their professional schedules. It was possible to complete all 216 home visits, across the three health posts, and to conduct the screening intervention co-developed with the CHWs during the classroom sessions.

Table 3 presents the responses provided by CHWs to the pre-training self-assessment. It is notable that their average assessments of their own knowledge about a range of related issues were low, ranging from an average Likert score of 1.1 for intubated feeding/medicating to 2.2 for recognising signs of elder abuse. The same pre-course assessment also asked CHWs what they considered to be the most important challenges associated with caring for older people and their suggested responses (Table 4). It is notable that the most frequently identified problems related to the capacity and willingness of family members to meet older people’s general care needs and therefore this issue was given a prominent focus in the training.

**Table 3:** CHWs’ self-assessment of knowledge related to older people’s health before and after the training intervention

Characteristic	Pre course, *N* = 55^1^	After course, *N* = 50^1^	*p*-value^2^
Health post			
GM	20 (36%)	17 (34%)	0.9
JEH	13 (24%)	13 (26%)	
JP	22 (40%)	20 (40%)	
Prevention of pressure sores			
0	10 (19%)	0 (0%)	< 0.001
1	20 (37%)	4 (8.0%)	
2	20 (37%)	8 (16%)	
3	4 (7.4%)	33 (66%)	
4	0 (0%)	5 (10%)	
Intubated feeding or intubated administration of medicine			
0	25 (45%)	4 (8.0%)	< 0.001
1	17 (31%)	13 (26%)	
2	11 (20%)	17 (34%)	
3	2 (3.6%)	13 (26%)	
4	0 (0%)	3 (6.0%)	
Body hygiene			
0	3 (5.5%)	0 (0%)	< 0.001
1	18 (33%)	2 (4.0%)	
2	27 (49%)	5 (10%)	
3	7 (13%)	25 (50%)	
4	0 (0%)	18 (36%)	
Characteristic	Pre course, *N* = 55^1^	After course, *N* = 50^1^	*p*-value^2^
Identification of possible signs of infection			
0	9 (16%)	1 (2.0%)	< 0.001
1	29 (53%)	5 (10%)	
2	13 (24%)	12 (24%)	
3	4 (7.3%)	24 (48%)	
4	0 (0%)	8 (16%)	
Identification of potential abuse			< 0.001
0	3 (5.5%)	1 (2.0%)	
1	16 (29%)	2 (4.0%)	
2	17 (31%)	11 (22%)	
3	17 (31%)	25 (50%)	
4	2 (3.6%)	11 (22%)	

^1^n (%)

^2^Kruskal-Wallis rank sum test

Source: Survey data

**Table 4:** CHW (*n* = 55) responses about the main challenges associated with caring for older people and suggested responses

Challenge	Number of responses	% of CHWs	Suggested reponse	Number of responses	% of CHWs
Insufficient care	37	61,67	Improve attention from family health team	32	53,33
Lack of family willingness to care	24	40,00	Capacity building for family care-givers	31	51,67
Uncertainty about care needs	23	38,33	More homes visits by doctors and nurses	24	40,00
Lack of patience by carer	22	36,67	Less red tape for obtaining care materials	16	26,67
Poor socio-economic situation	11	18,33	Support for family/care-giver	15	25,00
Lack of home visits by doctors or nurses	10	16,67	Address health problems of the care-giver	14	23,33
Lack of care materials	9	15,00	Improve CHW knowledge about dependent older people	10	16,67
Dificulty acessing tests	7	11,67	Better communication with families	10	16,67
Family lack knowledge about care-giving	7	11,67	Improve conditions in the home environment	6	10,00
Attention from health post/family health team	7	11,67	More priority from government	3	5,00

Source: Survey data

It was possible to compare CHWs’ assessments of their knowledge about different older people’s health issues before they took part in the course and afterwards (Table 3). For all these issues, there were significant *p*-value increases in their assessed knowledge. For all issues other than intubated feeding/medication, 60% or more of responses were 4 (good) or 5 (very good). The lower scores for this issue indicate a possible need for further training of this practice. It is also noteworthy that even after the training 12% of participants rated their knowledge of how to identify signs of common infections as poor or very poor, although this is a considerable improvement on the pre-course scores (69%).

More generally, the CHWs’ evaluations of the course itself were unanimously positive in terms of the usefulness of both the class-based sessions and the subsequent home visits. Some suggested that the number of class hours could be increased in order to increase their knowledge of and confidence in dealing with more complex and technically challenging issues.

Table 5 shows data on older peoples’ activities of daily living (ADLs) obtained from the home visit screenings. The most frequently reported ADL restriction was transferring (ability to get in and out of a bed chair, or wheelchair), which affected 71% of older people. The least frequent was independent feeding (39%). Table 6 presents data on risk factors obtained from the home visits. It is notable that in only five out of 192 visits were no risk factors identified. This indicates the degree of vulnerability of this population, the capacity of trained CHWs to categorise it and (potentially) inform other local health professionals about needs for specific forms of support.

**Table 5:** Number and proportion of older people identified by CHWs as unable to perform specific activities of daily living

Unable to independently	Number of older people visited	%
Transfer unaided	153	70,83
Go to toilet unaided	136	62,96
Bathe self	121	56,02
Continent	99	45,83
Dress self	90	41,67
Feed self	85	39,35
All of the above	28	12,96

Source: Survey data

**Table 6:** Risk factors for dependent older people included in the training programme and subsequently identified by CHWs (*n* = 50) during home visits

	Number of older people visited	% of older people visited
Risk of fall	144	66,67
Sleeping during the day	119	55,09
Care-giver overburdened	117	54,17
Difficulty in falling or staying asleep at night	114	52,78
Problems with memory and/or confusion about place or time	106	49,07
“Moaning” or other indications of pain	86	39,81
Difficulty swallowing or risk of choking	56	25,93
Pressure marks on the skin	50	23,15
Urine with a “fetid smell” (suggesting urinary infection)	49	22,69
4 or more days since defecation	46	21,30
Reduced consumption of food/drink	30	13,89
Scarring to the skin	22	10,19
Intubated feeding	5	2,31
No risk factor	5	2,31

Source: Survey data

The classroom interaction between CHWs and the trainers provided some illustrative, albeit unsystematic, indications of how their understanding of the needs of dependent older people and their care-givers was enhanced. For example, in one session a CHW shared the experience of a local woman who was experiencing “carer-burnout” related to looking after her mother who had severe dementia and without assistance from other family members. In a later session, the same CHW commented she had come to realise that some aspects of this person’s care burden could be alleviated simply by showing her more suitable ways to care for people with dementia. Similarly, a different CHW was initially very critical of a family’s care-giving for a highly dependent older person. In a later session, she commented that by being less judgemental and by listening to family members own problems, she had been able to develop a more effective care partnership with them.

## Discussion

This paper presents an innovative training intervention which seeks to enhance the capacity of CHWs to meet new health challenges which are rapidly emerging in low and middle-income countries such as Brazil. As populations age, the need for focussed training for CHWs related to the needs of care dependent older adults is self-evident. The limited reach of formal social care services in cities like Fortaleza increases the importance of effective collaborations between primary health care providers and families. Training CHWs and buttressing their role within these collaborations will be an essential part of reconfiguring health services towards a new social and epidemiological context.

As well as findings specifically related to the intervention, the study provides a range of other insights of relevance to the potential role of CHWs in supporting dependent older people. The emphasis CHWs gave to insufficient family care for dependent older people when identifying the main issues affecting dependent older people is in keeping with the findings of studies across Brazil [2, 26]. It is telling that in 117 out of 192 visits, CHWs reported the over-burden of family care-givers. This justifies the inclusion of training components that consider the wellbeing and capacity of family care-givers in interventions of this type [27]. Similarly, the high proportion of dependent older people who were seen as at risk of a fall may reflect the nature of the specific environment (crowded and substandard housing in deprived neighbourhoods) as much as it does their intrinsic status.

As well as enabling CHWs to take a more systematic approach to supporting older people and their families, their engagement with vulnerable older people in the community could provide the basis for more proactive and effective support from other parts of the city’s health and social care systems. Evidence from similar community-based interventions for older people in other parts of Brazil demonstrate that they can lead to more effective use of health services and reductions in potentially avoidable hospitalisations [26]. In Brazil urinary tract infection (UTI) is the leading cause of avoidable hospitalisation of people aged 60 and over [28]. In the Fortaleza pilot, CHWs identified 49 potential cases of poorly controlled UTIs. If this were reported back to the health centre and addressed in a timely way, there would be opportunities to prevent acute health episodes.

A key indication of the potential of any pilot intervention is what happens after it comes to an end and whether it generates lasting positive legacies. It had been planned to conduct follow-up sessions with the CHWs and repeat the house visits 6 months after the pilot was completed. This, however, coincided with the first weeks of the COVID-19 pandemic in Fortaleza, which has been one of the worst-affected cities in Brazil to date. In August 2020, the department of health agreed to fund a new project. This builds on and extends the original pilot intervention, will include a larger number of neighbourhoods and will establish a more formal structure of cooperation between health and social assistance agencies, such as regular joint case review of highly vulnerable older people. CHWs will play a central part in this new intervention, making repeat home visits to older people and families identified as high risk. This new project should offer new insights for LMICs about how to meet the needs of fast-growing numbers of dependent older people.

There are a number of important limitations in this study, mainly reflecting the small-scale of the pilot intervention and, as a result, the limited amount of data it generated. Although the study allowed for some comparison over time, it does not constitute a formal impact evaluation. Additionally, despite the anonymity of the evaluation process, it is possible that the positive responses about learning outcomes was biased by a desire to please those responsible for the training programme. Nevertheless, the results indicate that interventions of this kind may generate a range of important potential benefits in these settings.

## Conclusion

This study demonstrates the feasibility of developing interventions to seek to enhance the capacity of CHWs to meet the needs of dependent older people and their care-givers in countries like Brazil. The participant self-assessments indicate significant improvements in their perceived knowledge and capacity in responding to the health needs of care-dependent older people. Additionally, participants were able to successfully conduct the home visits and screening for risk factors. This evidence of effectiveness, though limited and subjective, provides justification for larger, formally evaluated interventions. No studies of similar interventions have been published for Brazil or other LMICs. Consequently, the experience of Fortaleza offers valuable lessons for other cities and countries which are facing similar challenges.

## Data Availability

The datasets used and/or analysed during the current study are available from the corresponding author on reasonable request.

## Abbreviations

ADL: Activities of daily living
CHW: Community health worker
DOP: Dependent older people
LMIC: Low and middle-income countries
NCD: Non-communicable disease
PCU: Primary care unit
SUS: Unified Health System

## Footnotes

1 Full ethical clearance was provided by the Ethics Committee of the School of International Development, University of East Anglia, UK.
